# Influence of contact pressure, cross-shear and counterface material on the wear of PEEK and CFR-PEEK for orthopaedic applications

**DOI:** 10.1016/j.jmbbm.2016.06.005

**Published:** 2016-10

**Authors:** C.L. Brockett, S. Carbone, A. Abdelgaied, J. Fisher, L.M. Jennings

**Affiliations:** Institute of Medical and Biological Engineering, School of Mechanical Engineering, University of Leeds, Leeds, UK

**Keywords:** Orthopaedics, Wear, PEEK, CFR-PEEK, Pin-on-plate

## Abstract

Total joint replacement is a successful surgical intervention for the treatment of the degeneration of many joints, particularly the hip and knee. As the demand for joint replacement grows, and the life expectancy of the population increases, the performance requirements of these implants also changes. New materials, to improve longevity and enhance performance have been explored including PEEK and CFR-PEEK.

This study investigated whether CFR-PEEK and PEEK were appropriate materials for total joint replacement by examining wear performance in simple configuration studies articulating against cobalt chrome under a range of cross-shear and contact pressure conditions. Simple geometry pin on plate studies were conducted for one million cycles for each test condition, with the contact pressure and cross-shear conditions representing a range in which the material may need to operate in-vivo.

The wear factor for PEEK was significantly higher than CFR-PEEK and conventional polyethylene under all test conditions. Both PEEK and CFR-PEEK wear were influenced by contact pressure, with the highest wear factors for both materials measured at the highest pressure conditions. PEEK appeared to have a cross-shear dependent wear response, but this was not observed for the CFR-PEEK material.

This study has further characterised the wear performance of two materials that are gaining interest for total joint replacement. The wear performance of the PEEK material showed poorer wear performance compared to polyethylene when articulating with a metal counterface, but the performance of the CFR-PEEK material suggested it may provide a suitable alternative to polyethylene in some applications. The wear performance of CFR-PEEK was poorer than polyethylene when it was used as the plate, when there was translation of the contact zone over the surface of the CFR-PEEK plate. This has implications for applications in low conforming contacts, such as lower conformity knee replacement.

## 1 Introduction

Total joint replacement has been a successful surgical intervention for joint degeneration for decades. Each year more than 90 000 replacement hips and knees are implanted in the UK alone (12th Annual Report National Joint Registry for England, 2015). Hip and knee replacements are both considered successful surgical procedures, with less than 10% failure within the first ten years of implantation for most designs (Mäkelä, 2014, Engh et al., 2012, Argenson et al., 2013). However, there is increasing demand for joint replacement in younger and more active patients. The majority of total joint replacements utilise an ultra-high molecular weight polyethylene (UHMWPE) bearing, articulating with either a metallic or ceramic counterface. It is well documented that the wear of polyethylene releases small particles into the surrounding tissue that elicit a dose-dependent osteolytic response (Ingham and Fisher, 2000, Tipper et al., 2000, Fisher et al., 2009). In more active patients, it may be expected they may reach the osteolytic threshold earlier than less active patients. In order to improve implant longevity and reduce wear, alternative bearing materials have been sought.

Carbon-fibre reinforced poly ether-ether ketone (CFR-PEEK) and poly ether-ether ketone (PEEK) have been used for some time in spinal cages and bone fixation devices (Kurtz and Devine, 2007). In the last decade, these materials have been explored through pre-clinical studies as bearing surfaces for total joint replacement. PEEK is a linear thermoplastic material, with an aromatic spine and ketone and ether functional units (Kurtz, 2011). Similar to UHMWPE, it is semi-crystalline meaning it has both amorphous and crystalline regions. It has good thermal and chemical stability, and is known to be resistant to damage from radiation, hence can be readily sterilised (Kurtz, 2011).

A number of studies have been conducted evaluating both PEEK and CFR-PEEK materials for joint replacement application. Pin-on-plate and pin-on-disk studies have been used for many years to screen materials, and explore the influence of parameters such as lubricant, sliding distance, contact pressure and cross-shear ratio on the wear of orthopaedic materials (Kang et al., 2008, Saikko, 1998, Saikko and Ahlroos, 2000, Cooper et al., 1993, Barbour et al., 1995). Pin-on-plate studies examining PEEK and CFR-PEEK to date have used polymer pins articulating with metal or ceramic plates, and have generally demonstrated CFR-PEEK to have equivalent or superior wear properties than UHMWPE under comparable conditions (Scholes and Unsworth, 2009, Scholes and Unsworth, 2007). It is notable that these studies have not examined the performance of these materials under a broad range of conditions. There appear to have been fewer studies conducted with unfilled PEEK articulating with a hard counterface, but more recently self-mating bearing couples and PEEK on UHMWPE have been explored (Grupp et al., 2010, Scholes and Unsworth, 2010). All demonstrated potential application for PEEK as a bearing surface when articulating with another polymer, with promising wear results. Notably, there was indication that CFR-PEEK on UHMWPE was not an appropriate combination due to elevated polyethylene wear resultant from the abrasive nature of the carbon-fibres (East et al., 2015).

Full joint simulation has explored CFR-PEEK bearing materials in uni-compartmental knee replacements (Grupp et al., 2010, Scholes and Unsworth, 2009), conventional hip replacement (Wang et al., 2012, Brockett et al., 2012) and a novel horse-shoe cup for total hip replacement (Scholes et al., 2008). The knee wear studies, based on highly conforming low contact stress implant designs in current clinical use, both demonstrated pitch-based CFR-PEEK against cobalt-chromium (CoCr) femoral bearings to have lower or equivalent wear performance to UHMWPE (Grupp et al., 2010, Scholes and Unsworth, 2009). Hip simulation has been conducted using CFR-PEEK acetabular cups articulating with ceramic (Biolox Delta or Forte) femoral heads. All studies indicated significantly lower wear rates in the CFR-PEEK bearings compared with UHMWPE (Wang et al., 2012, Brockett et al., 2012, Scholes et al., 2008). There was some concern expressed regarding the high friction measured compared with conventional material combinations (Brockett et al., 2012, Flanagan et al., 2010) but all studies indicated the bearing as a promising material for application in total hip replacement. Wang et al. (2012) studied the effect of cup inclination angle, and, for the range studied, identified that the wear of CFR-PEEK was not sensitive to cup positioning. However, it was not clear whether the contact reached the edge of the cup and hence whether edge loading occurred.

It is evident from the experimental literature to date that PEEK and CFR-PEEK have potential as bearing materials for total joint replacement, however, as other authors have indicated, a more in-depth understanding of the factors influencing wear performance is required. The aim of this study was to characterise and compare the wear properties of PEEK and CFR-PEEK articulating against a CoCr counterface under a range of cross-shear and contact pressure conditions, to explore the potential application of these materials for total joint replacement. As previous studies had also indicated the arrangement of the counterface surfaces may influence wear, this was also explored.

## 2 Materials

**Fig. 1:**
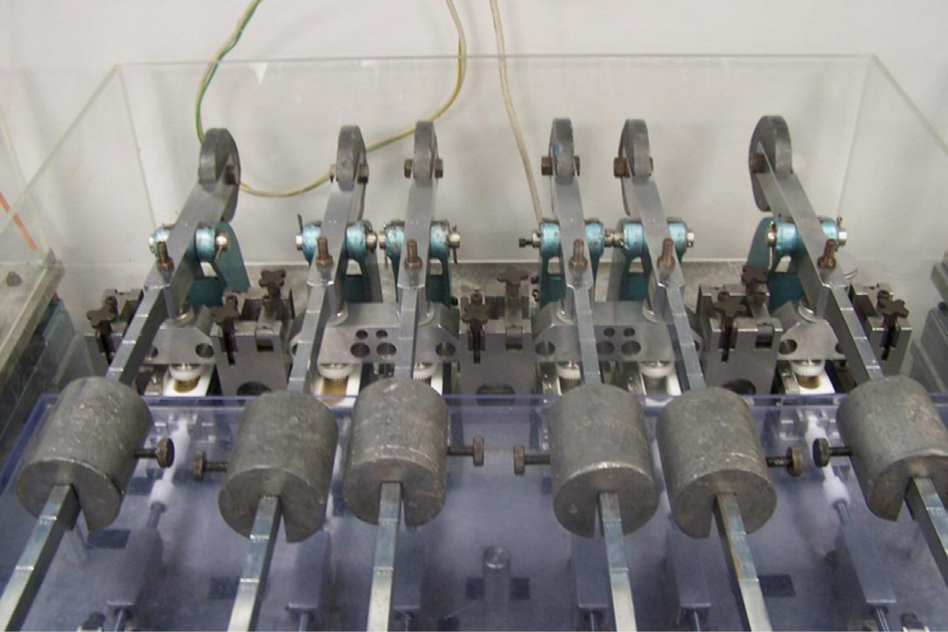
Pin on plate rig.

Several studies were conducted to examine the wear characteristics of PEEK and CFR-PEEK (Table 1). PEEK (Optima, Invibio) and CFR-PEEK (Motis, Invibio) materials were injection moulded and machined into pins forming a truncated cone, with a flat-faced articulating surface of 3, 5, or 8 mm diameter, and an external diameter of 12 mm. The orientation of the pitch-based carbon fibres (30% fill by weight) within the CFR-PEEK material was not controlled during the manufacturing process. PEEK, CFR-PEEK and GUR1020 UHMWPE materials were machined into flat plates of 58 mm long, 25 mm wide and 5 mm deep. These were used in conjunction with bespoke fixtures to allow them to be tested in the pin-on-plate rig (Fig. 1). Following manufacture of the polymeric pins and plates, all samples were placed in deionised water for a minimum of 12 weeks, in order for the fluid uptake to stabilise (Wang et al., 2012).

Medical grade high carbon cobalt chromium (CoCr) plates were polished and lapped until they had a smooth surface finish (Ra ~0.01 µm). Medical grade high carbon CoCr pins were machined with a spherical articulating surface of 35 mm radius (external pin radius of 12 mm) and polished until they had a smooth surface finish.

## 3 Methods

A multidirectional pin on plate wear simulator (University of Leeds) was used to determine the wear factors of PEEK and CFR-PEEK articulating against cobalt chrome under a range of conditions (Kang et al., 2008). The pin specimens were placed in a collet and loaded with a constant load throughout the study, and the plate was reciprocated beneath the pin. A rack and pinion gear mechanism was used, with a rack fitted to the side of the bath, and the gear attached to the pin collet (Fig. 1). The combination of pin rotation and plate translation resulted in multidirectional motion at the contacting surface (Kang et al., 2008; Abdelgaied et al., 2013). The pin rotation and plate translation were in phase with a common frequency of 1 Hz, and the motions linked such that the pin rotation was zero at the centre of the stroke length, and at a maximum at the end of the stroke.

Several studies were designed to explore the influence of contact pressure, cross shear and configuration upon the wear of PEEK and CFR-PEEK under conditions representative of in-vivo in total hip or knee replacement. The contact pressure and cross-shear ratio conditions were selected to be comparable to previously reported studies on polyethylene tested in the same pin on plate simulators (Kang et al., 2008, Abdelgaied et al., 2013, Galvin et al., 2006).

Each study was conducted for a period of one million cycles (Mc), and wear was assessed gravimetrically at 0.33Mc intervals. Testing was conducted in 25% (v/v) new born bovine serum (16 g/L protein concentration), supplemented with 0.03% (v/v) sodium azide solution to retard bacterial growth. Throughout all studies, two unloaded soak control samples for each condition were immersed in the same lubricant, and stored within the simulator, such that they were exposed to identical temperature and lubrication conditions. At each measurement point, the control samples and the pins and plates were removed from the fixtures and cleaned accordingly to an established in-house protocol before the samples were allowed to air dry in a controlled environment for 72 h prior to weighing (Brockett et al., 2012). The components were weighed using Mettler Toledo AT21 balance (Leicester, UK, resolution 1 µg). Gravimetric measurements were converted to volumetric wear using a density for CFR-PEEK of 1.42 g/cm^3^, for PEEK of 1.3 g/cm^3^ and for UHMWPE of 0.94 g/cm^3^. The wear factor, *k*, was calculated using the following equation (Galvin et al., 2006):

$$k=\frac{V}{PX}$$

where *k* is the wear factor (mm^3^/Nm), *V* is the volumetric wear (mm^3^), *P* is the applied load (N) and *X* is the sliding distance (m).

Surface roughness measurements of the plates were recorded at the start and completion of each study to examine the changes in counterface over the duration of the study. A contacting profilometer (Talysurf, Taylor Hobson, UK) was used to take five traces perpendicular to the direction of sliding across the plate surfaces. A least-squares line form removal, and a Gaussian filter of 100:1 bandwidth, with a cut-off of 0.25 mm was used to allow the roughness to be analysed.

### 3.1 Study 1: influence of contact pressure on wear of PEEK and CFR-PEEK

The first study examined the influence of contact pressure on the wear of the PEEK and CFR-PEEK materials. Each test was conducted with a stroke length of 26 mm and a rotation of ±45°, resulting in an average cross shear condition of 0.18 (Kang et al., 2008). An applied load of 80 N was used for each study, giving contact pressures of 1.6 MPa, 4 MPa, and 11 MPa for the 8 mm, 5 mm and 3 mm diameter pin counterfaces respectively. Six pins of each material were tested against CoCrMo plates, and mean wear factors calculated for each condition.

### 3.2 Study 2: influence of cross-shear on the wear of PEEK and CFR-PEEK

The effect of cross shear on the wear of PEEK and CFR-PEEK was undertaken through adjusting the stroke length and rotation conditions for each study (Table 2). The range of cross shear ratio conditions studied was from 0 (unidirectional motion) to 0.254 (multi-directional motion), representing the range of conditions which might occur at the bearing interface in total joint replacement. The pin counterface for all studies was 5 mm, with an 80 N constant load, to give a mean contact pressure of 4 MPa. A minimum of five pins (maximum of six) were tested for each material against CoCrMo plates.

### 3.3 Study 3: influence of counterface arrangement

The effect of counterface arrangement was explored by using PEEK, CFR-PEEK and GUR1020 UHMWPE plates articulating with CoCrMo pins with a curved counterface of 35 mm radius. A curved pin surface was used to reduce the edge-effect of a metallic pin articulating with a polymer plate. Each test was conducted with a stroke length of 26 mm and a rotation of ±45°, resulting in an average cross shear condition of 0.18. A constant load of 80 N was applied throughout all studies. Six plates of each material were studied.

Statistical analysis was performed using one-way ANOVA, and a Tukey post-hoc analysis applied if statistical significance (*p*<0.05) was observed.

## 4 Results

**Fig. 2:**
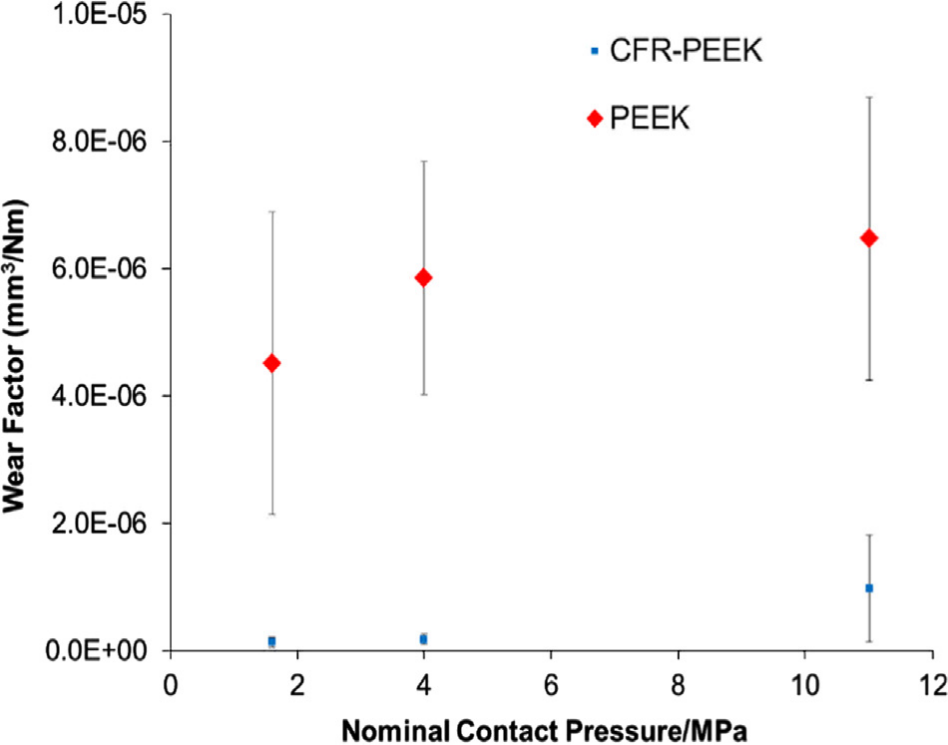
Influence of contact pressure on wear of PEEK and CFR-PEEK pins articulating with CoCr plates (±95% confidence limits).

The wear factors of the PEEK and CFR-PEEK pins under a range of different contact pressures, studied with a cross shear ratio of 0.18 are shown in Fig. 2. The wear factors for the PEEK pins, tested against CoCr plates were higher than the CFR-PEEK pins under all test conditions. The wear factors for both PEEK and CFR-PEEK appeared to increase with increasing contact pressure, and reducing contact area, however this was not statistically significant for either material (*p*=0.21 and *p*=0.09 respectively). The maximum wear factors for both materials were measured under 11 MPa conditions, and were (6.38±2.54)×10^−6^ mm^3^/Nm and (1.19±0.66)×10^−6^ mm^3^/Nm for PEEK and CFR-PEEK respectively. The wear performance of the PEEK pins appeared to be more variable for each contact pressure compared with the CFR-PEEK pins; and the wear performance of CFR-PEEK seemed to become more variable under the high contact pressure conditions. It was notable that after the first 0.33Mc stage of testing, the counterface of the PEEK pins had worn such that the contact area was much larger for the following stages, but this did not appear to have an influence on wear over the duration of the study.

**Fig. 3:**
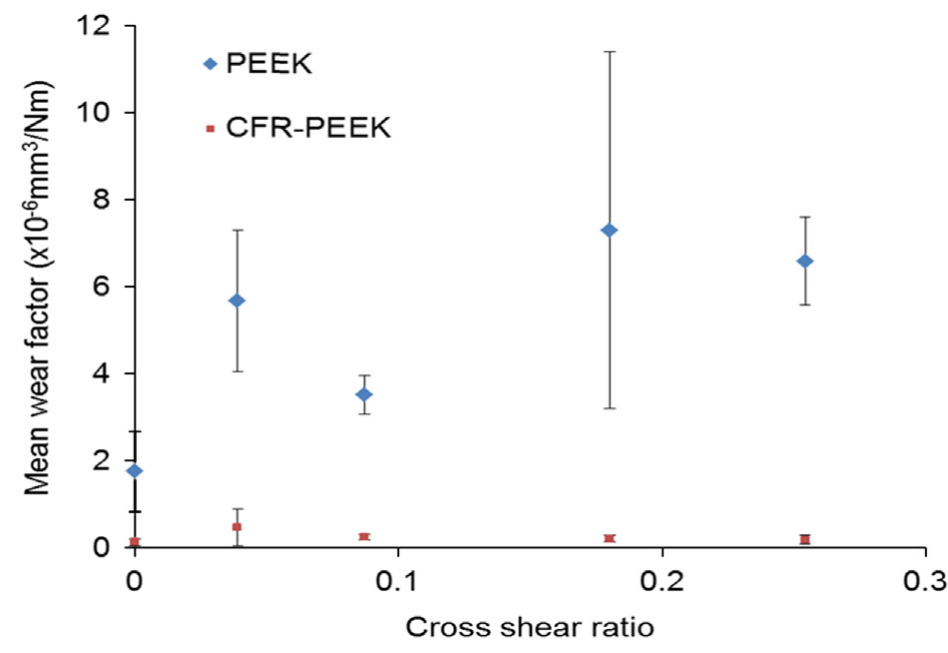
Influence of cross-shear on wear of PEEK and CFR-PEEK pins articulating with CoCr plates (±95% confidence limits).

The influence of cross-shear (CS) was explored by altering the sliding distance and rotation of the pin/plate combination from a unidirectional motion (CS ratio: 0; 28 mm/0°) to a multi-directional motion (CS ratio: 0.254; 38 mm/±55°). The wear factors for the PEEK and CFR-PEEK materials were both lowest for the unidirectional motion study (Fig. 3). The cross shear appeared to have little influence on the wear performance of the CFR-PEEK material, with the mean wear factors being similar for all cross-shear conditions. The PEEK material appeared to demonstrate cross-shear dependent wear behaviour, the lowest wear factor for PEEK, during unidirectional motion was (1.76±2.29)×10^−6^ mm^3^/Nm, increasing to a maximum of (7.29±2.18)×10^−6^ mm^3^/Nm at a cross-shear ratio of 0.18. Again, it was notable that the PEEK samples showed more variability during testing than the CFR-PEEK pins.

The mean surface roughness of the CoCrMo plates prior to testing was comparable for the PEEK and CFR-PEEK studies at 0.01 µm. At the completion of the cross-shear studies, the mean surface roughness of the plates had increased to 0.016 ± 0.013 µm and 0.026 ± 0.029 µm for the PEEK and CFR-PEEK tested CoCr plates respectively. There was no statistically significant difference between the two bearing combinations at the end of the study (*p*>0.05).

**Fig. 4:**
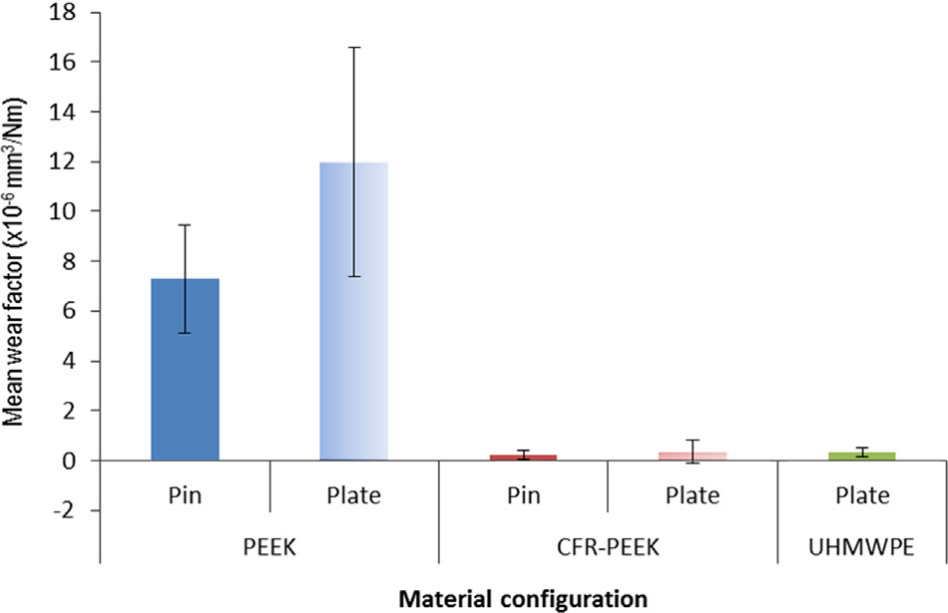
Effect of counterface configuration on wear factor (±95% confidence limits).

**Fig. 5:**
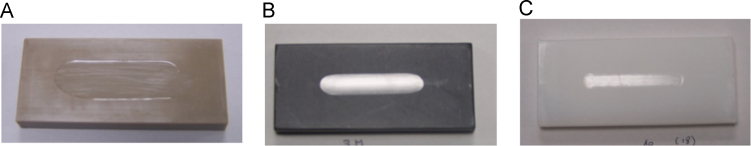
Comparison of wear scars on polymer plates after 1Mc (A: PEEK, B: CFR-PEEK, C: UHMWPE).

The influence of counterface arrangement was examined by testing CoCr pins with a curved articulating surface against machined flat PEEK, CFR-PEEK and UHMWPE plates, to explore whether the arrangement influenced the relative performance of the polymer materials. Six plates of each material were tested, and the difference in wear performance was demonstrated through the gravimetric analysis (Fig. 4), and the visual inspection of the wear scars (Fig. 5). The mean wear factor for the PEEK plates (11.97±4.69)×10^−6^ mm^3^/Nm was higher than both the CFR-PEEK and UHMWPE materials. The mean wear factor of the UHMWPE material ((0.15±0.16)×10^−6^ mm^3^/Nm) was lower than the CFR- PEEK material ((0.37±0.11)×10^−6^ mm^3^/Nm). This difference in wear performance was reflected in the wear scars observed at the end of the study, with the PEEK plates having wider and longer scars (mean length 35.0 mm, width 11.3 mm) than both the CFR-PEEK (mean length 31.0 mm, width 6.3 mm) and UHMWPE (mean length 32.8 mm, width 5.3 mm) plates. The effect of counterface was investigated by comparing the plate wear factors, with the pin wear factors tested under comparable loading conditions (Fig. 4). However, it should be noted due to the curvature of the metallic pin, the contact pressure conditions were not identical. The counterface arrangement had a significant effect on the PEEK material (*p*=0.048), but did not have such an effect on the CFR-PEEK material. In both materials, the wear factor for the metal pin on polymer plate was higher.

There was a difference between surface roughness of the polymeric plates at the start of the study, this was due to the mechanical properties of the polymeric materials and the manufacturing processes for the plates. The CFR-PEEK and PEEK plates were injection moulded, giving a smoother surface than the machined UHMWPE plates. The UHMWPE plates had the highest mean surface roughness of 2.29±1.25 µm, and the PEEK plates had the lowest mean roughness of 0.13±0.05 µm. The mean surface roughness of the CFR-PEEK plates prior to study was 1.04±0.11 µm. At the completion of the study, the mean surface roughness of the PEEK plates had increased significantly to 0.49±0.15 µm. The mean surface roughness of both the CFR-PEEK and UHMWPE plates had decreased to 0.76±0.11 µm and 1.76±0.99 µm respectively (*p*>0.05).

## 5 Discussion

The influence of contact pressure on the wear of PEEK and CFR-PEEK articulating with a metallic counterface was examined through three contact pressure conditions, by altering the cross-sectional area of the pin counterface under the same applied load. There was a non-statistically significant increase in wear factor with increasing contact pressure for both materials. This is in contrast to the widely-reported behaviour of UHMWPE, which has been shown to have reducing wear factors with increasing contact pressure (Abdelgaied et al., 2013, Galvin et al., 2009). A recent study exploring the influence of contact pressure on the wear of CFR-PEEK against an alumina counterface showed a similar trend, with the wear factors of CFR-PEEK at high contact pressure in the present study being comparable with those reported (Evans et al., 2014). The present study builds upon the previous work (Evans et al., 2014) as it explores a different bearing combination, and has tested sufficient samples to allow statistical analysis.

This is an important observation for translation of these materials for use in total joint replacement, when considering design and functional performance. Several studies have shown the low wear potential of a CFR-PEEK hip cup articulating with a ceramic head (Wang et al., 2012, Brockett et al., 2012, Scholes et al., 2008). In these designs, under ‘standard’ gait conditions, and optimal alignment, the contact pressure for the implant would be relatively low, and therefore the wear performance of these bearings is better than an equivalent polyethylene design. However, the findings from the present study and Evans et al. (2014) indicate that under higher contact stress conditions – for example adverse loading conditions such as edge loading, this benefit may be less apparent. Wang et al. explored the influence of cup angle on the wear of ceramic-on-CFR-PEEK, up to an inclination of 60° and found this to have no significant effect on the wear performance of the implant (Wang et al., 2012). However, the authors did not comment on whether the angle was sufficiently high to cause contact between the head and rim of the cup and hence edge loading, therefore it is not clear on whether these elevated cup angles would have generated higher stress conditions.

Application of CFR-PEEK to partial knee replacement has been explored experimentally (Grupp et al., 2010, Scholes and Unsworth, 2009), and shown reduced wear compared to conventional polyethylene inserts, but both studies investigated highly conforming designs, which would lead to lower contact pressure conditions than more conventional total knee replacement designs. Our findings would suggest the need for exploring these materials in total knee replacement design under more challenging conditions, such as high flexion activities including stair climb and squatting.

**Fig. 6:**
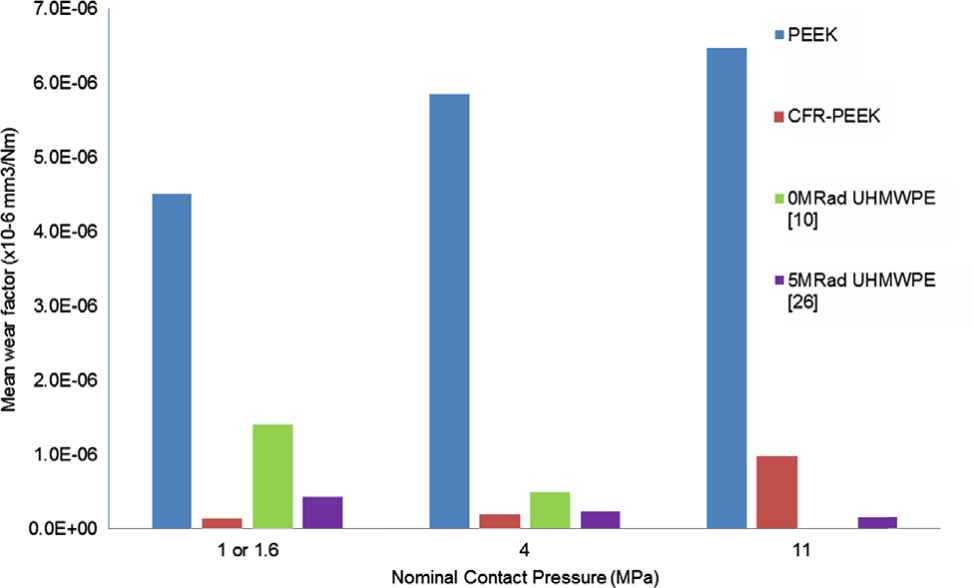
Comparison of wear factors with existing literature for UHMWPE (note previous studies were conducted at 1 MPa (Kang et al., 2008; Abdelgaied et al., 2013).

The wear of PEEK was higher than CFR-PEEK for all contact pressures, and conventional and moderately cross-linked polyethylene under comparable test conditions (Kang et al., 2008, Abdelgaied et al., 2013) [Fig. 6]. It should be noted that previous studies on polyethylene were conducted at 1 MPa and the present study was conducted with 1.6 MPa due to a difference in the size of pin counterface diameter. All studies were conducted within the same research laboratories under otherwise identical conditions. The comparison appears to suggest PEEK would offer no advantage in total joint replacement over polyethylene when articulating with a hard counterface, such as cobalt chromium. However, other recent studies, exploring PEEK articulating as either a self-mating couple or against polyethylene, as an alternative to a metallic bearing, have shown promise (Grupp et al., 2010, East et al., 2015, Cowie et al., 2014).

The influence of cross-shear on the wear of PEEK and CFR-PEEK was explored through several combinations of displacement and rotation, to create five cross-shear ratio conditions (Kang et al., 2008). Fig. 3 demonstrates that changing the cross-shear conditions had little effect on the wear factor of the CFR-PEEK, in contrast to the cross-shear dependency that has been noted in both conventional and cross-linked polyethylene (Kang et al., 2008, Abdelgaied et al., 2013). Unfilled PEEK material may undergo a molecular orientation in the principle direction of motion (strain hardening), which whilst enhancing wear performance in that direction, diminishes wear resistance in the perpendicular plane, hence increasing wear in conditions with higher cross-shear (Haward, 1993). Conversely, the presence of randomly oriented carbon fibres within the CFR-PEEK material prevents such a re-orientation and thus the material shows no cross-shear wear dependency. This independence may prove advantageous under more adverse wear conditions under higher kinematic demand.

Barbour et al. (1995) highlighted that the difference in polymer-metal surface interaction in a pin on plate study depends on the counterface orientation. In a study employing polymer pins articulating with a metallic counterface under a constant load condition, the polymer contact surface is constantly under stress. In the metal pin on polymer plate arrangement, the wear track of the polymer plate undergoes cyclic loading and unloading, thus two different wear conditions are created by using both material arrangements. It has already been demonstrated that for comparable conditions in the polymer-pin/metal-plate arrangement, PEEK had a much higher wear rate than CFR-PEEK and UHMWPE (conventional and cross-linked), and CFR-PEEK had a lower wear rate than conventional UHMWPE, and similar to cross-linked UHMWPE (Fig. 6). When the arrangement was changed to metal pin on polymer plate, the mean wear factor for the PEEK material was the highest measured in all studies. The wear factor for the CFR-PEEK was higher than any of the polymer pin/metal plate studies, and also higher than the wear factor measured for the metal pin articulating with conventional UHMWPE. The change in counterface for polyethylene, when compared with previously published polymer pin/metal plate studies, appears to have had the least impact on wear factor. It is important to consider the influence of these test conditions on the relative wear performance of the materials, and how this may translate to a total joint replacement.

## 6 Conclusions

This study explored the wear performance of PEEK and CFR-PEEK materials under a range of conditions as potential materials for total joint replacement. The wear of PEEK against metallic counterface was higher than CFR-PEEK, and previously reported polyethylene materials, and therefore this would indicate that PEEK would not be an advantageous alternative for polyethylene in polymeric/metal bearing combinations. Under several test conditions, the wear performance of CFR-PEEK appeared to be favourable with respect to UHMWPE, and the wear was not dependent on cross-shear. However, the increase in wear with contact pressure, and the increase in wear of CFR-PEEK when subject to translation of the contact would indicate further need to explore non-conforming contacts and adverse conditions and design considerations in the knee pre-clinically before applying this material to a total knee joint replacement.

## Open data

The data associated with this paper (surface roughness and wear study data) are openly available from the University of Leeds Data Repository (Brockett et al., 2016).

## Figures and Tables

**Table 1:** Pin and plate materials and geometry for each study.

Study	Plate materials	Pin materials	Pin counterface (mm Φ)
Contact Pressure	CoCr	PEEK/CFR-PEEK	Flat 3, 5, 8
Cross Shear	CoCr	PEEK/CFR-PEEK	Flat 5
Counterface	1020GUR UHMWPE/PEEK/CFR-PEEK	CoCr	Curved, 35

**Table 2:** Cross shear conditions.

Cross shear ratio	Rotation ( ° )	Stroke length (mm)
0	0	20
0.039	±20	20
0.087	±30	28
0.18	±45	26
0.254	±55	38
